# Movement of small RNAs in and between plants and fungi

**DOI:** 10.1111/mpp.12911

**Published:** 2020-02-06

**Authors:** Mengying Wang, Ralph A. Dean

**Affiliations:** ^1^ Fungal Genomics Laboratory Center for Integrated Fungal Research Department of Entomology and Plant Pathology North Carolina State University Raleigh NC USA

**Keywords:** cross kingdom, extracellular vesicles, small RNAs, transportation

## Abstract

RNA interference is a biological process whereby small RNAs inhibit gene expression through neutralizing targeted mRNA molecules. This process is conserved in eukaryotes. Here, recent work regarding the mechanisms of how small RNAs move within and between organisms is examined. Small RNAs can move locally and systemically in plants through plasmodesmata and phloem, respectively. In fungi, transportation of small RNAs may also be achieved by septal pores and vesicles. Recent evidence also supports bidirectional cross‐kingdom communication of small RNAs between host plants and adapted fungal pathogens to affect the outcome of infection. We discuss several mechanisms for small RNA trafficking and describe evidence for transport through naked form, combined with RNA‐binding proteins or enclosed by vesicles.

## Graphical abstract

We summarize the evidence and possible mechanisms that small RNAs move within and between plants and fungi in naked form, combined with RNA‐binding proteins or enclosed in vesicles.

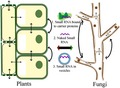

**Abbreviations**
AGO: Argonaute
CC: companion cell
CW: cell wall
CYP: cytochrome P450 lanosterol C‐14 α‐demethylase
DCL: dicer‐like
DM: desmotubule
dsRNA: double‐stranded RNA
EHMx: extrahaustorial matrix
ER: endoplasmic reticulum
EV: extracellular vesicle
FCW: fungal cell wall
FIGS: filamentous organism‐induced gene silencing
FPM: fungal plasma membrane
G: Golgi
GFP: green fluorescent protein
HIGS: host‐induced gene silencing
miRNA: microRNA
MP: movement protein
MVB: multiple vesicle body
N: nucleus
PCM: plant plasma membrane
PCW: plant cell wall
PD: plasmodesmata
piRNA: PIWI‐interacting RNA
PM: plasma membrane
RBP: RNA‐binding proteins
RDRP: RNA‐dependent RNA polymerase
RNAi: RNA interference
RNPC: ribonucleoprotein complex
SC: source cell
SE: sieve tube element
siRNA: small interfering RNA
SP: sieve tube plate or septal pore

## 1 INTRODUCTION

Small RNAs were first discovered in *Escherichia coli* in 1984 (Mizuno *et al.*, 1984). Subsequently, they have been found in all kingdoms of life operating as noncoding RNA with diverse functions (Wassarman *et al.*, 2001; Saito, Kakeshita, and Nakamura, 2009; Pantaleo *et al.*, 2010; Li *et al.*, 2016). Most small RNAs serve as regulators of gene expression (Hammond *et al.*, 2000; McCaffrey *et al.*, 2002; Paul *et al*., 2002). In eukaryotes, small RNAs induce silencing of target genes, known as RNA interference (RNAi), at both transcriptional and post‐transcriptional levels. Here, their defining features are short length (c.20–30 nucleotides) and association with proteins of the Argonaute family, with whose help they can recognize target mRNAs and lead to their reduced expression (Ghildiyal and Zamore, 2009). Based on origin, they are typically classified as small interfering RNA (siRNA), microRNA (miRNA), and PIWI (P‐element‐induced wimpy testes)‐interacting siRNA (piRNA; Ghildiyal and Zamore, 2009).

siRNA, miRNA as well as piRNA all act to control gene expression and play important roles in many fundamental biological processes in eukaryotic organisms. They have been tied to vital processes such as cell growth, tissue differentiation, heterochromatin formation, cell proliferation, and disease resistance (Blair and Olson, 2015; Yuan *et al.*, 2015; Tassetto *et al.*, 2017; Czech *et al.*, 2018; Mondal *et al.*, 2018; Almeida *et al.*, 2019). Research over the past few decades has led to powerful insight into the structure and function of small RNAs, which has been summarized in several reviews (Eamens *et al.*, 2008; Ghildiyal and Zamore, 2009; Peters and Meister, 2007; Pratt and MacRae, 2009; Holoch and Moazed, 2015; Quinn and Chang, 2016; Zhang, Cozen *et al.*, 2016; Zhang *et al.*, 2019). The purpose of this review, however, is to highlight what is known and not known about the mechanisms of how small RNAs move within and between organisms. Indeed, small RNAs can travel both short and long distances in plants, as well as in fungi. Below, we summarize their movement in plants and fungi before considering how small RNAs move between fungi and plants.

## 2 SHORT‐ AND LONG‐DISTANCE MOVEMENT OF SMALL RNAS IN PLANTS AND FUNGI

In plants, small RNAs are produced to coordinate plant development, maintain genome integrity, and combat adverse environmental conditions (Buchon and Vaury, 2006; Chen, 2009; Ruiz‐Ferrer and Voinnet, 2009). The mobility of small RNAs was presumed to be a prerequisite for carrying out these functions. Evidence now shows that small RNAs can move both short and long distances in plants (Sarkies and Miska, 2014). Primary siRNA can spread 10–15 cells without producing secondary siRNA (Kim, 2005), while long‐distance small RNA movement involves amplification of silencing signals through RNA‐dependent RNA polymerases (RDRPs) that are transported primarily through the phloem (Wassenegger and Krczal, 2006). Transitivity and secondary siRNA production amplify the RNAi so silencing persists even in the absence of the initiator double‐stranded RNA (dsRNA)(Baulcombe, 2004). As early as 1928, Wingard found the upper leaves of a tobacco plant whose lower leaves had been inoculated with tobacco ringspot virus and showed strong symptoms became resistant to the same virus (Wingard, 1928). We now know that the recovery from virus disease involves small RNAs derived from the virus moving from the infection site to upper leaves and conferring small RNA‐mediated resistance in the distal tissues (Ratcliff, 1997; Baulcombe, 2004).

### 2.1 Cell‐to‐cell (short‐range) movement in plants

**Figure 1:**
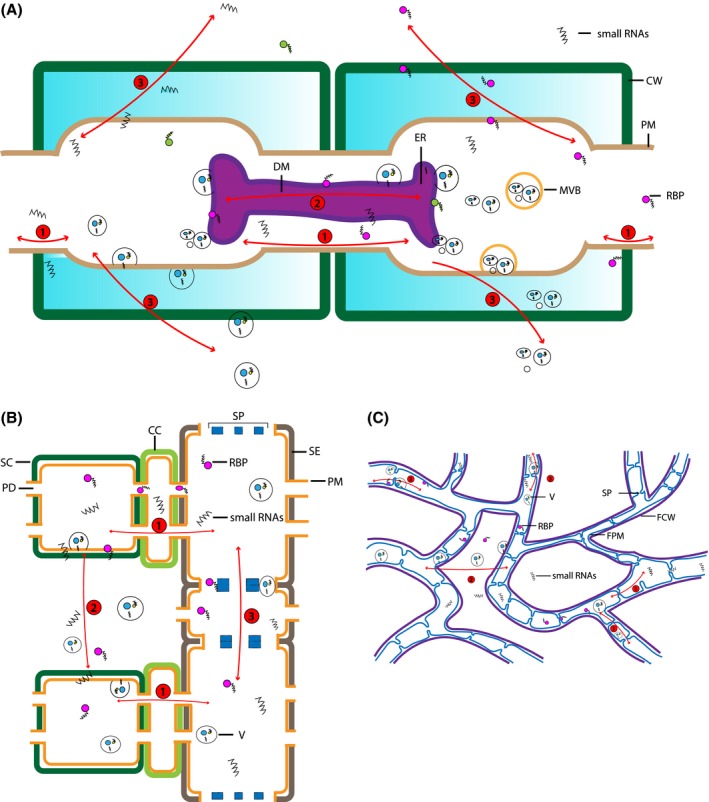
Short‐ and long‐distance transportation of small RNAs in plants and fungi. (a) Cell‐to‐cell movement in plants: 1, naked small RNAs, small RNAs bound to RNA‐binding proteins (RBP), and small RNAs enclosed in vesicles can move from cell to cell through spaces between the plant plasma membrane (PM) and desmotubule (DM); 2, small RNAs can be transported through the DM, which connects the endoplasmic reticulum (ER) of two adjacent cells; 3, small RNAs can be secreted from the PM and travel through the plant cell wall (CW) to extracellular spaces, and small RNAs can also be taken up by other cells (multiple vesicle bodies, MVB). Note: Vesicle transport through plasmodesmata by active gating is hypothetical at this time. (b) Long‐distance movement in plants: 1, naked small RNAs, small RNAs bound to RBP, and small RNAs inside vesicles can be transported from source cells (SC) to companion cells (CC) and then to sieve tube elements (SE) through plasmodesmata; 2, small RNAs can be secreted out of PM and travel through the plant cell wall (CW) to extracellular spaces and subsequently be absorbed by other cells; 3, small RNAs can be transported to distal plant cells through the sieve tube elements (sieve tube plates, SP). (c) Movement in fungi: 1, naked small RNAs, small RNAs bound to RBP, and small RNAs inside vesicles can be transported short distances cell to cell through the septal pore (SP); 2, small RNAs can be secreted out of fungal plasma membrane (FPM) and travel through the fungal cell wall (FCW) to extracellular spaces. Later, small RNAs can be absorbed by distal fungal cells and in this way small RNAs can be dispersed systemically throughout the whole fungal colony; 3, small RNAs can be transferred through the FPM. Unlike nonselective transportation through septal pores, FPM can conduct selective transportation by binding, fusion, and secretion. Note: small RNA movement in fungi needs more evidence.

The early clear evidence for mobile small RNAs was reported using *Nicotiana benthamiana* plants expressing the *GFP* transgene. Leaf infiltration with *Agrobacterium* also expressing *GFP* resulted in a ring of *GFP* silencing that was consistently observed spreading over 10–15 cells beyond the agroinfiltration zone without triggering small RNA amplification. When an RNA silencing suppressor was co‐infiltrated, *GFP* silencing was abolished (Johansen, 2002). In addition to siRNA generated by transgenes, endogenous miRNAs have also been observed to spread from cell to cell. For example, when miR390 precursor loci were transcribed in the vascular system and pith region of *Arabidopsis*, mature miR390 were found only in the shoot apical meristem and young leaf primordia where their precursors were not detected (Chitwood *et al.*, 2009). Similarly, miR165/166 precursors were transcribed mainly in the endodermis of *Arabidopsis* root, but mature miR166 were observed in adjacent cell layers (Carlsbecker *et al.*, 2010). These and other examples are consistent with the cell‐to‐cell movement of miRNA (Chitwood et al., 2009; Martínez et al., 2016; Wu and Zheng, 2019) (see Figure 1a).

### 2.2 Long‐range movement in plants

Long‐range systemic movement was first demonstrated by Dalmay in 2000 using a phloem‐restricted virus expressing a *GFP* reporter gene. The virus was applied to *GFP* expressing plants and *GFP* silencing was observed for entire leaves (Dalmay *et al*., 2000). Later, Pant and colleagues demonstrated the long‐range movement of miRNA through micrografting *Arabidopsis* plants. In grafted plants with miR393 overexpressing shoots and wild‐type roots, high levels of miR393 accumulated in the roots, suggesting the long‐range movement (shoot to root) of miR393 (Pant *et al.*, 2008). Molnar demonstrated that both exogenous and endogenous small RNAs could pass through the graft union (Molnar *et al.*, 2010). Other studies using grafted *Nicotiana tabacum* as well as *Arabidopsis* showed small RNAs can transfer from source tissue (leaves) to meiotically active cells such as anthers and flowers (Zhang *et al.*, 2014; see Figure 1b).

### 2.3 Movement of small RNAs in fungi

In contrast to plants, fungi are simple organisms and lack defined cellular transportation systems for the movement of nutrients and metabolites. Fungi may exist as unicellular forms or as extensive multicellular hyphal branched networks. A number of fungi, including Zygomycota, are usually aseptate; in contrast, other fungal divisions like Ascomycota and Basidiomycota hyphae are separated by septa, which usually have pores. Small RNAs have also been well characterized in fungi (Drinnenberg *et al.*, 2009; Nicolas *et al.*, 2010; Nunes *et al.*, 2011; Mueth *et al.*, 2015; Campo *et al.*, 2016; Donaire and Ayllón, 2017). In 1992, small RNAs were first demonstrated to mediate gene silencing, termed quelling, in *Neurospora crassa* (Romano and Macino, 1992). Subsequently, similar phenomena were reported in many fungal phyla, including Ascomycetes and Basidiomycetes, as well as in fungal‐like Oomycota (Nicolás, Torres‐Martínez, and Ruiz‐Vázquez, 2003; Latijnhouwers *et al.*, 2004; Wang *et al.*, 2010; Nunes *et al.*, 2011). Studies of the direct movement of small RNAs within fungal colonies and tissues are largely absent. However, transfection of protoplasts with dsRNA can lead to targeted gene silencing that is maintained for several months across a growing colony, suggestive of both amplification and movement (Caribé dos Santos *et al.*, 2009; Saraiva *et al.*, 2014).

## 3 TRANSPORTATION PATHWAYS OF SMALL RNAS IN PLANTS AND FUNGI

Conceptually, molecules, including small RNAs, can be transported between cells and tissues within an organism via two principal routes: through direct internal connections (symplast) or externally (apoplast). In either case, evidence exists that small RNAs can be transported either in naked form or encased in vesicles (Bucher *et al.*, 2001; Cai *et al*., 2018a; Kehr and Buhtz, 2008; Koch *et al.*, 2016; Vogler *et al.*, 2008).

### 3.1 Transport as either a naked form or encased in vesicles

Evidence for transport of naked forms is primarily inferred from direct application of small RNAs to cells. Both plants and fungi, including fungal‐like oomycetes, have the capacity to import naked small RNAs. Whisson and colleagues described the first application of transient gene silencing by delivering in vitro synthesized dsRNA directly into protoplasts of the oomycete *Phytophthora infestans* to trigger silencing (Whisson *et al.*, 2005). Similar gene silencing results were observed using the basidiomycete *Moniliophthora perniciosa*, which causes witches’ broom disease on cacao. In this instance, protoplast transfection with synthesized dsRNA led to targeted gene silencing for as long as 4 months after dsRNA treatment (Caribé dos Santos *et al.*, 2009). Production of secondary siRNA may occur to amplify the silencing effect and small RNAs may move through the whole fungal colony. In *Saprolegnia parasitica*, dsRNA‐mediated long‐term gene silencing has also been reported (Saraiva *et al.*, 2014). Moreover, when artificial synthesized siRNA were co‐cultured with the model filamentous fungus *Aspergillus nidulans*, silencing of the reporter *GFP* gene as well as endogenous *AnrasA* & *B* genes was induced, supporting the possibility that this may be a natural means of small RNA transport in fungi (see Figure 1c) (Kalleda *et al.*, 2013).

Direct application of RNA molecules to plants has been shown to down‐regulate endogenous transcript levels. Sammons *et al*. (2011), in a patent application, showed that direct application of various nucleic acids, including dsRNA and siRNA, down‐regulated herbicide resistance (Sammons *et al*., 2011). Through root soaking, dsRNA targeting *Mob1A* and *WRKY23* was delivered into *Arabidopsis* and rice tissue. Suppression of root growth, seed germination, and failure of bolt or flower were detected along with silencing of the targeted genes (Li *et al.*, 2015). Besides suppression of plant endogenous genes, a number of studies have demonstrated that direct application of dsRNAs can effectively silence transgenes such as *GFP* or *YFP* in plants (Dubrovina *et al.*, 2019).

As an alternative to the naked form, small RNAs can also be transported through a pathway involving vesicular migration from the endoplasmic reticulum (ER) to the Golgi apparatus and then loading to a complex network of vesicles. Small RNAs can be sorted to transporting vesicles fusing with the plasma membrane and then released by exocytosis (Bonifacino and Glick, 2004). Compared to plants, knowledge of vesicular transport in fungi is extensive. Such extracellular vesicles (EVs) have been discovered in many different species of fungi, such as *Cryptococcus neoformans*, *Histoplasma capsulatum*, *Candida albicans*, *Candida parapsilosis*, *Sporothrix schenckii*, and *Saccharomyces cerevisiae* (Albuquerque *et al.*, 2008; Rodrigues *et al.*, 2008, 2007). In addition to proteins, neutral lipids, glycans, and pigments, fungal RNA has also been found in EVs (Rodrigues *et al.*, 2007; Oliveira *et al.*, 2010, 2009; Vallejo *et al.*, 2012; Garcia‐Silva *et al.*, 2014). Different types of noncoding small RNAs have been characterized inside EVs from *C. neoformans*, *Paracoccidiodes brasiliensis*, and *C. albicans* as well as from *S. cerevisiae* (Da Silva *et al.*, 2015).

**Figure 2:**
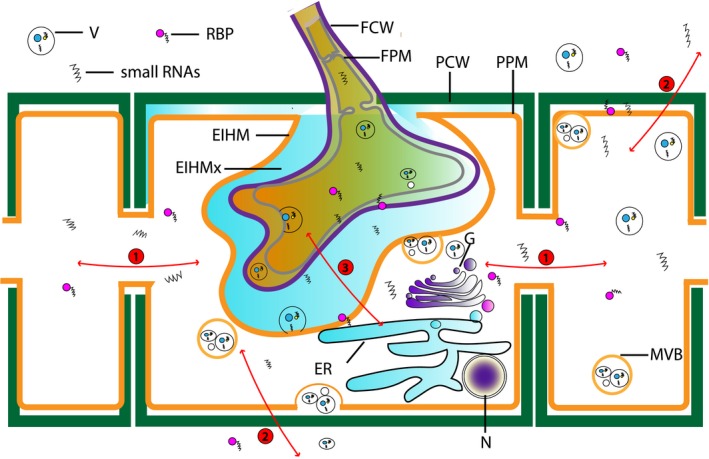
Trans‐kingdom transportation of small RNAs between plants and fungi: 1, inside plant cells, naked small RNAs, small RNAs bound with RBP, and small RNAs inside vesicles can be transported cell to cell through plasmodesmata (PM); 2, small RNAs can be secreted through the plant plasma membrane (PPM) and plant cell wall (PCW) to extracellular spaces, where they can also be taken up by other cells; 3, small RNAs can be transferred through the fungal plasma membrane (FPM)–fungal cell wall (FCW)–extra‐invasive hyphae matrix (EIHMx)‐extra‐invasive hyphae membrane (EIHM)–plant cytoplasm pathway. This transportation pathway can be bidirectional. N, nucleus; G, Golgi. Note: Vesicles transport through the plasmodesmata by active gating is a hypothesis.

As each cell has two endomembrane systems, one for outgoing traffic and the other for incoming traffic (Hilbi and Haas, 2012), small RNAs can be released from the cell through EVs as well as be absorbed by the recipient cell through membrane fusion. This has been demonstrated using synthetic EVs composed of siRNA inside cationic lipid/liposomes (Spagnou *et al.*, 2004). Moreover, the trafficking of EVs by fungal cells is regulated by both cell turgor and cell wall structure (Eisenman *et al.*, 2005; Brown *et al*., 2015). Thus, the fungal cell wall may play an important role in regulating the movement of small RNAs (via EVs) between fungal cells and to plant hosts (Figure 2).

### 3.2 Movement of small RNAs via the symplast and apoplast

In plants, for movement through the symplast, small RNAs probably move through the plasmodesmata (PD), a plasma membrane‐lined pore acting as an intercellular channel that connects the plant cytoplasm of connected cells (Figure 1a). There are several lines of evidence supporting the symplast route. Mature guard cells that are symplastically isolated from adjacent cells escape transitive *GFP* silencing (Voinnet *et al.*, 1998; Vatén *et al.*, 2011). The presence of the tobacco mosaic virus movement protein (MP) increased PD aperture size and enhanced the spread of transgene silencing (Bucher *et al.*, 2001; Vogler *et al.*, 2008). Several viruses transfer their RNA genome to plant cells through ER protrusions that extend through the PD (Chou *et al.*, 2013; Pyott and Molnar, 2015).

The PD size exclusion limit is 30–50 kDa and may dictate which forms of small RNAs can move through the PD. Naked small RNAs are around 15 kDa, thus their free diffusion through the PD should not be limited (Crawford and Zambryski, 2000). As the size of plant vesicles (>10 nm) (Huang *et al.*, 2017; Rutter and Innes, 2017) is generally larger than the diameter of PD microchannels (3–4 nm) (Ding *et al.*, 1999; Sager and Lee, 2018), vesicles containing small RNAs may not diffuse freely through the PD. However, PD permeability can be significantly increased through dilation, active gating, and structural remodelling (Lucas and Lee, 2004). Thus, naked as well as vesicle‐enclosed small RNAs may be trafficked across the PD actively in plants.

Several lines of evidence indicate that long‐range movement of small RNAs is primarily by means of the phloem (Figure 1b). In 2008, Buhtz identified a large number of small RNAs in the phloem of oilseed rape plants but not in the xylem. Analysis of phloem sap contents revealed different types of RNAs (Kehr and Buhtz, 2008), while for xylem exudates only minerals, peptides, and proteins were found (Turnbull and Lopez‐Cobollo, 2013). In 2010, Varkonyi and co‐workers also found a subset of miRNAs present in the phloem of apple (Buhtz *et al.*, 2008; Varkonyi‐Gasic *et al.*, 2010). In addition, Roberts showed that treatment of plants with a nontoxic concentration of cadmium to block phloem transport of specific virus movement also inhibited systemic RNA silencing (Ghoshroy *et al.*, 1998; Ueki and Citovsky, 2001). In vascular plants, phloem is a living tissue that conveys organic compounds made during photosynthesis from source (typically leaves) to sink tissues (such as roots and buds) (Van Bel, 2003). However, in several solanaceous species as well as *Arabidopsis*, upward long‐distance mobile silencing has also been shown to be phloem mediated (Liang *et al.*, 2012).

Proteins may assist in both short‐ and long‐range transportation of naked small RNAs. In plants, RNA‐binding proteins (RBPs), which are at the core of ribonucleoprotein complexes (RNPCs), are important for RNA movement (Kedde *et al.*, 2007). Phloem Small‐RNA Binding Protein 1 (CmPSRP1) from pumpkin (*Cucurbita maxima*) phloem binds single‐stranded small RNAs moving from cell to cell through the PD (Yoo *et al.*, 2004). This protein can also shuttle small RNAs through the companion cell–sieve element complex (Ham *et al.*, 2014). Several small RBPs have been identified in the phloem of other plant species (Barnes *et al.*, 2004; Go, 2004; Giavalisco *et al.*, 2006). Argonaute (AGO) proteins have also been suggested to be involved in siRNA movement (Marín‐González and Suárez‐López, 2012).

For fungal cells that are linked to each other, intercellular communication may be achieved via septal pores, similar to plasmodesmata in plant cells (Bloemendal and Kück, 2013). Septal pores were first reported by Bary in 1884 (Bary, 1884). Later, in 1893, Wahrlich observed cytoplasmic flow between different fungal compartments (Wahrlich, 1893). Septa can be described as a simple plate with a central pore about 50–500 nm in diameter that allows the passage of cytoplasm and organelles like mitochondria, vacuoles, and nuclei (Gull, 1978; Esser, 1982). Moreover, microtubules have also been found to direct the transport process in filamentous fungi and the range of cargo can be expanded to include endosomes, mRNA, peroxisomes, and secretory vesicles (Egan *et al.*, 2012). It was further demonstrated that tubules can move cargo in either direction across the septal pores as well as transport material between cells (Shepherd *et al.*, 1993). In sum, the septal pore, a plasmodesmata‐like structure associated with ER or the desmotubule, a membranous cell wall‐spanning structure, may enable small RNAs either in naked form or enclosed in vesicles to move throughout the whole mycelial network (Zarnack and Feldbrügge, 2007; Figure 1c).

Transport via symplastic routes is probably valuable for movement within an organism and where direct cellular connections exist. For the apoplastic pathway, small RNAs are proposed to be exported by the cell through the membrane to the apoplast and are subsequently imported into a recipient cell, potentially another organism in intimate proximity. Direct evidence demonstrating the apoplastic transportation pathway for small RNAs within plants is lacking. However, the discovery of diverse small RNA species in *Arabidopsis* extracellular vesicles is consistent with this pathway where small RNAs could be loaded into vesicles and secreted to the apoplast (Baldrich *et al.*, 2019). Extracellular vesicles play critical roles in fungal growth and the ability to derive nutrients from their environment (including invading potential hosts). Recently, a number of different types of noncoding small RNAs have been characterized inside EVs from fungi as described above (Garcia‐Silva *et al.*, 2014; Peres da Silva *et al.*, 2015; Rayner *et al.*, 2017).

## 4 CROSS‐KINGDOM TRAFFICKING OF SMALL RNAS

Insight gained into the conservation of mechanisms of RNA silencing and understanding the movement of small RNAs within different organisms opens up the distinct possibility that small RNAs could be readily shared between organisms in close association to induce gene silencing. Bidirectional small RNA movement between host and parasite was first reported in 2012 between the honeybee and *Varroa destructor* (Garbian *et al.*, 2012). Today the evidence suggests that cross‐kingdom RNAi can occur between diverse living systems (Roney *et al.*, 2006; David‐Schwartz *et al.*, 2008; Lamonte *et al.*, 2012; Cheng *et al.*, 2013; Garcia‐Silva *et al.*, 2014; Weiberg *et al.*, 2014; Quintana *et al.*, 2015).

### 4.1 Endogenous small RNA transfer between plants and fungi

The role of cross‐kingdom RNAi for defining interactions between fungal pathogens and plant hosts was pioneered by Hailing Jin's group. They showed that to promote virulence, the necrotrophic fungal pathogen *Botrytis cinerea* produces small RNAs during infection that hijack the host plant's RNAi machinery to silence genes of *Arabidopsis* and tomato involved in host immunity. *B. cinerea dcl1 dcl2* double mutants that could no longer produce Bc‐sRNA exhibited reduced virulence, whereas the *Arabidopsis ago1* mutant that lost RNAi function regained resistance to *B. cinerea*. This kind of host plant gene silencing triggered by small RNAs from the fungus has been termed filamentous organism‐induced gene silencing (FIGS; Baulcombe, 2015; Weiberg *et al.*, 2014).

The trafficking of small RNAs is bidirectional; plants can also deliver endogenous small RNAs into invading fungal pathogens. For example, miR166 and miR159 generated in cotton have been shown to be transferred to the hyphae of the wilt pathogen *Verticillium dahliae* during infection, where they reduced expression of genes encoding a Ca^2+^‐dependent cysteine protease (*Clp‐1*) and an isotrichodermin C‐15 hydroxylase (*HiC‐15*). Deletion of those two genes in the fungus inhibited microsclerotia formation or hyphae growth, respectively, and down‐regulation of *Clp‐1* and *HiC‐15* through small RNAs from the host plant interfered with fungal pathogenicity (Zhang *et al.*, 2016). A growing number of recent studies suggest that both plants and fungi use cross‐kingdom RNAi strategies for their own benefit (Table 1).

**Table 1:** Summary of small RNAs movement between plants and fungi

Plant host	Fungal life style	Fungal pathogen	Target genes	Evidence	Reference
Barley	Biotrophic	*Blumeria graminis*	Effector gene *Avra10*	Reduced fungal development	Nowara *et al.* (2010)
Barley	Biotrophic	*B. graminis*	50 *Blumeria* effector candidates	Eight were identified contributing to infection	Pliego *et al.* (2013)
Wheat	Biotrophic	*Puccinia striiformis* f. sp*. tritici*	Calcineurin homologs *Pscna1/Pscnb1*	Slower extension of fungal hyphae and reduced production of urediospores	Zhang *et al.* (2012)
Wheat	Biotrophic	*P. striiformis* f. sp*. tritici*	MAPK kinase gene *PsFUZ7*	Hyphal development strongly restricted, necrosis of plant cells in resistance responses induced	Zhu *et al.* (2017)
Wheat	Biotrophic	*P. striiformis* f. sp*. tritici*	PKA catalytic subunit gene *PsCPK1*	Significant reduction in the length of infection hyphae and disease phenotype	Qi *et al.* (2018)
Wheat	Biotrophic	*Puccinia triticina*	MAP kinase (*PtMAPK1*), cyclophilin (*PtCYC1*), and calcineurin B (*PtCNB*)	Disease suppression, compromising fungal growth and sporulation	Panwar *et al.* (2013a)
Wheat	Biotrophic	*P. triticina*	Three predicted pathogenicity genes encoding MAPK, cyclophilin, and calcineurin regulatory subunit	Suppressed disease phenotype	Panwar *et al.* (2013b)
Wheat	Biotrophic	*Puccinia graminis* f. sp*. tritici*	Haustoria‐enriched genes	Reduced fungi development	Yin *et al.* (2015)
Lettuce	Biotrophic oomycete	*Bremia lactucae*	*Highly abundant message #34* (*HAM34*), *cellulose synthase* (*CES1*)	Greatly reduced growth and inhibition of sporulation	Govindarajulu *et al.* (2015)
Potato	Biotrophic oomycete	*Phytophthora infestans*	Three genes important in the infection, *PiGPB1, PiCESA2*, and *PiPEC*, together with *PiGAPDH* taking part in basic cell maintenance	Hp‐PiGBP1 targeting the *G protein β‐subunit* (*PiGPB1*) important for pathogenicity resulted in most restricted disease progress	Jahan *et al.* (2015)
Potato	Biotrophic oomycete	*P. infestans*	RXLR effector *Avr3a* gene	Imparted partial resistance to late blight disease	Sanju *et al.* (2015)
*Arabidopsis*, barley	Hemibiotrophic	*Fusarium graminearum*	Fungal *cytochrome P450 lanosterol C‐14α‐demethylase* (*CYP51*) genes	Inhibition of fungal growth	Koch *et al.* (2013)
Banana	Hemibiotrophic	*Fusarium oxysporum* f. sp. *cubense*	*Velvet*, *Fusarium transcription factor 1*	Resisted disease at 8 months post‐inoculation	Ghag *et al.* (2014)
*Arabidopsis*	Hemibiotrophic	*F. oxysporum*	*F‐box protein required for pathogenicity 1* (*FRP1*), *F. oxysporum Wilt 2* (*FOW2*), *plant 12‐oxophytodienoate‐10,11‐reductase gene* (*OPR*)	Survival rates after fungal infection were higher in the transgenic lines	Hu *et al.* (2015)
Wheat	Hemibiotrophic	*F. graminearum*	*Chitin synthase* (*Chs*) *3b*	High levels of stable, consistent resistance to both fusarium head blight and fusarium stem blight throughout the T_3_ to T_5_ generations	Cheng *et al.* (2015)
Wheat	Hemibiotrophic	*F. graminearum*	β‐1,3‐glucan synthase gene *FcGls1*	Aberrant, swollen fungal hyphae	Chen, Kastner *et al.* (2016)
*Arabidopsis*, barley	Hemibiotrophic	*F. graminearum*	*CYP51* genes	Spray‐induced gene silencing also conferred resistance against *F. graminearum* in unsprayed distal leaf parts	Koch *et al.* (2016), Wang and Jin (2017)
Wheat, barley	Hemibiotrophic	*F. graminearum*	*TRI6*, a transcription factor that positively regulates deoxynivalenol synthesis	Silencing of *TRI6*	Hunter *et al*. (2018)
Cotton	Hemibiotrophic	*Verticillium dahliae*	Two *V. dahliae* genes encoding a Ca^2+^‐dependent cysteine protease (*Clp‐1*) and an isotrichodermin C‐15 hydroxylase (*HiC‐15*)	Cotton plants increased production of microRNA 166 (mir166) and mir159 that silence *Clp‐1* and *hic‐15*	Zhang *et al.* (2016)
Cotton	Hemibiotrophic	*V. dahliae*	*V. dahliae* *hygrophobins1 *(*VdH1*) gene	Induced silencing of the target mRNA and conferred resistance to *V. dahliae* infection	Zhang *et al.* (2016)
*Arabidopsis,* tomato	Hemibiotrophic	*V. dahliae*	Three previously identified virulence genes of *V. dahliae* (*Ave1, Sge1,* and *NLP1*)	Reduced verticillium wilt disease in two of the three targets	Song and Thomma (2018)
*Arabidopsis*, tomato	Necrotrophic	*Botrytis cinerea*	*B. cinerea* Dicer‐like protein encoding genes: *Bc‐DCL1* and *Bc‐DCL2*	Silenced *Bc‐DCL* genes and attenuated fungal pathogenicity and growth	Weiberg *et al.* (2014), Wang *et al.* (2016)
*Arabidopsis*	Necrotrophic	*B. cinerea*	small RNAs‐containing vesicles accumulate at the infection sites and are taken up by the fungal cells	Transferred host sRNAs induced silencing of fungal genes critical for pathogenicity	Cai *et al.* (2018)
Tall fescue	Necrotrophic	*Rhizoctonia solani*	Genes encoding RNA polymerase, importin beta‐1 subunit, Cohesin complex subunit Psm1, and a ubiquitin E3 ligase	Lesion size was reduced by as much as 90%	Zhou *et al.* (2016)
Tobacco	Necrotrophic	*Sclerotinia sclerotiorum*	*Chitin synthase* (*Chs*)	Reduction in disease severity	Andrade *et al.* (2016)
Maize	Saprotrophic	*Aspergillus* species	*AflC* gene encodes an enzyme in the *Aspergillus* aflatoxin biosynthetic pathway	Aflatoxin could not be detected	Thakare *et al.* (2017)

### 4.2 HIGS: artificial small RNAs transfer from plants to fungi

Observations that naturally occurring endogenous small RNAs move between organisms led to studies that showed that artificial transgene‐derived small RNAs are also able to move between interacting organisms. This has been exploited for the development of host‐induced gene silencing (HIGS), a novel RNA‐based technology for the efficient control of fungal pathogens and other pests (see Table 1). Conceptually, HIGS involves generating small RNAs targeting a pathogen gene in the host plant, which results in the uptake of small RNAs and gene silencing in the invading pathogen. HIGS has been demonstrated in a number of diverse fungal pathosystems and provides a promising disease control alternative to chemical control (Nowara *et al.*, 2010; Yin *et al.*, 2011; Zhang *et al.*, 2012; Panwar *et al.*, 2013; Hu *et al.*, 2015; Deising *et al*., 2016; Song and Thomma, 2018; Zhang *et al*., 2016; Zhou *et al.*, 2016; Zhu *et al*., 2017; Qi *et al*., 2018). In addition, it also can be used as a tool to screen potentially crucial fungal genes without the need to produce knockout mutants, which is challenging in a number of pathogens (Yin *et al.*, 2015).

Small RNAs have been shown to transfer bidirectionally between plants and fungi; however, the mechanism(s) of how they move remains to be fully determined.

### 4.3 Possible pathways for small RNA cross‐kingdom movement

Based on studies of small RNA movement in plants and fungi described above, there are several pathways for cross‐kingdom small RNA transportation. Because naked small RNAs can move short and long distances in plants (Hyun *et al.*, 2011) and can also be taken up by fungal cells (Wang, Thomas, and Jin, 2017), trafficking during plant–fungus interactions may involve naked small RNAs. For instance, when small RNAs targeting *B. cinerea DCL1* and *DCL2* genes were directly sprayed to *Arabidopsis* and tomato, treated plants gained resistance to grey mould disease, suggesting the naked exogenous small RNAs were assimilated into the pathogen and interfered with fungal virulence (Wang *et al.*, 2016). Furthermore, spraying dsRNA of the fungal *CYP3* gene on barley conferred resistance to *Fusarium graminearum* not only at the local sprayed area but also at distal nonsprayed areas. *CYP* encodes a protein required for fungal ergosterol synthesis, and silencing of this gene is lethal for fungi (Koch *et al.*, 2016). McLoughlin and colleagues applied 59 in vitro‐synthesized dsRNAs onto the leaf surface of oilseed rape and *Arabidopsis*, 20 of which suppressed disease symptoms caused by *Sclerotinium sclerotiorum* and *B. cinerea* along with reduced expression levels of target genes (Mcloughlin *et al.*, 2018). The number of examples of direct RNA molecule uptake leading to local and systemic resistance against fungal pathogens is growing (Wang *et al.*, 2016; Song *et al.*, 2018; Gu *et al.*, 2019). Direct application of small RNAs has been referred to as spray‐induced gene silencing (SIGS). In most studies, however, direct uptake is limited without tissue wounding (Song *et al.*, 2018). How these molecules are taken up and first assimilated to the plant before transfer to the pathogen remains to be determined. The process may be facilitated by RBPs in plants protecting small RNAs from degradation (McEwan *et al.*, 2012). However, there are no similar reports regarding small RNA‐binding proteins in fungi or of receptors on the fungal cell surface to recognize small RNAs. Thus, movement via membrane‐bound vesicles may better explain small RNA communication between plants and fungi.

For both plants and fungi, vesicles containing small RNAs can be generated inside cells as well as secreted to the extracellular environment. Evidence suggests that such vesicles can also be taken up by fungi (Jiang *et al.*, 2012; Brown *et al.*, 2015). Recently, *Arabidopsis* cells have been shown to secrete extracellular vesicles to deliver plant small RNAs into the fungal pathogen *B. cinerea*, resulting in silencing of fungal genes critical for pathogenicity (Cai *et al.*, 2018).

Studies of mammals such as mice also suggested that small RNAs can be transferred between different species mediated by EVs (Knip *et al.*, 2014). Such vesicles probably enable genetic communication between phylogenetic distantly related organisms. For example, plant‐derived exosome‐like nanoparticles have been detected in the guts of mice after consuming plant material. These ingested plant‐derived exosome‐like nanoparticles contain proteins, lipids, and small RNAs (Mu *et al.*, 2014). Direct evidence for vesicle involvement in plant–pathogen interactions has also been obtained in barley leaves under attack by powdery mildew pathogen *Blumeria graminis*. Light microscope‐visible vesicle‐like bodies were observed accumulating around papillae, which formed at sites where the fungal penetration was halted, suggesting such vesicles may be important for host immunity (An *et al.*, 2006). These vesicles are known to contain antimicrobial compounds, such as phytoalexins, phenolics or reactive oxygen species (Tam *et al.*, 2015). In addition, vesicle‐like inclusions have been shown to accumulate around penetration sites in sorghum leaves attacked by the hemibiotrophic fungus *Colletotrichum graminicola* (Nielsen *et al.*, 2004). In onion, membrane‐bound electron‐dense vesicles were observed in epidermal cells in response to necrotrophic fungus *Botrytis allii*. Furthermore, vesicle budding and fusion of vesicle‐like structures with the fungal plasma membrane have been observed in the *Arabidopsis–Golovinomyces orontii* interaction. Fungal multiple vesicle bodies (MVBs) were abundant in haustoria and putative exosome vesicles were detected in the extracellular space and extrahaustorial matrix (EHMx), suggesting the existence of an exosome‐mediated secretion pathway for the interaction area between plants and fungi. These and numerous other examples support a role of vesicles in plant–fungal pathogen interactions (Chowdhury, 2016; Stewart and Mansfield, 1985). Evidence to date also suggests vesicles derived from both plants and fungi can contain small RNAs. However, further research is required to confirm whether vesicles at the fungal–host plant interface do, indeed, contain small RNAs, and that they are released into the extracellular space and are subsequently taken up by the associated partner. The movement of EVs between organisms may be highly regulated and directed rather than occur by simple diffusion (see Figure 2).

Direct evidence for the role of vesicles in cross‐kingdom communication could be obtained through the isolation of vesicles derived from HIGS transgenic plants followed by evaluation of the presence of target small RNAs inside vesicles. Such vesicles could then be co‐cultured with fungi to confirm the ability to confer RNA silencing. Using fluorescence or radioactively labelled small RNAs would facilitate monitoring of small RNA movement. Chemical inhibitors such as Brefeldin A (Nebenfuhr *et al.*, 2002), prieurianin (Robert *et al.*, 2008; Tõth *et al.*, 2012), and secramine (Pelish *et al.*, 2006) that block vesicle secretion may be valuable to confirm the function of extracellular vesicles.

The possible mechanisms of small RNA absorption remain unknown. Secretion and absorption may involve cell membrane proteins. Currently, there are limited reports regarding protein channels for small RNA movement in plants. In addition, how specific small RNAs are sorted for secretion and absorption remain enigmatic. Though much work needs to be done, studies of small RNA communication will probably provide applications for enhancing sustainable agriculture. Gene silencing of pathogen genes by HIGS or the direct application of dsRNA, for instance, is a highly promising strategy to provide resistance to plant disease.

## 5 CONCLUSION

The RNAi system is largely conserved among eukaryotes. It plays important biological roles, including in disease processes. Both plants and fungi can generate small RNAs and induce gene silencing. In plants small RNAs can be transported both short and long distances. For the latter, small RNAs are transferred from cell to cell through plasmodesmata and the plant phloem. Small RNAs can be transferred nakedly, bound by proteins, or packed into vesicles. In fungi small RNAs can be transferred through septa pores as well as secreted and absorbed in vesicles. EVs are present at the interface of plant–fungus interactions. However, compelling data are still needed to illuminate the underlying mechanisms by which small RNA communication occurs across plants and fungal cells. The knowledge of how small RNAs are sorted and transferred to target cells is also unclear. Answers to these questions and others related to cross‐kingdom communication will not only enrich our understanding of plant disease processes but also aid in the development of powerful new tools for disease control.

## Data Availability

Data sharing is not applicable to this article as no new data were created or analysed in this study.
